# Anæsthesia from the Internal Use of Chloroform

**Published:** 1866-02

**Authors:** 


### ANÆSTHESIA FROM THE INTERNAL USE OF CHLOROFORM.

Noticing an article on Chloroform in the *Reporter* of October 28th, Dr. J. N. FREEMAN, of Morris Illinois, late Surgeon 106th N. Y. S. I., writes as follows ;

"On the Second of July last, at the Delevan House in Albany, my Brother, J. A. FREEMAN, M. D., who was with me, was attacked with cholera morbus, with crampings so severe as made him cry out with pain. I gave him various remedies without avail, till at last the urgency of the symptoms induced me to give him twenty-five drops of chloroform internally, and repeat the dose in about five minutes, when the anæsthetic effect was speedily manifested, rendering him perfectly insensible. Dr. J. V. P. QUACKENBUSH was present before he recovered from the stupor produced by the chloroform, and as the crampings partially returned, advised another dose of chloroform internally, which again produced its anæsthetic effect, after which there was but little return of the crampings, and we were able to resume our journey the next day."

